# Hyperaldosteronism Presenting as Pre-syncope: A Case Report

**DOI:** 10.7759/cureus.98735

**Published:** 2025-12-08

**Authors:** Michael Mazar, Ramin Tabibiazar, Ravi Dave

**Affiliations:** 1 Cardiology, University of California Los Angeles David Geffen School of Medicine, Los Angeles, USA

**Keywords:** acute flaccid muscle weakness, muscle weakness, pre-syncope, primary hyperaldosteronism, reversible cause of muscle weakness

## Abstract

Primary Aldosteronism (PA) is a common but often underrecognized cause of secondary hypertension. While typically associated with hypokalemia, profound muscle weakness is rarely a presenting symptom, especially in the absence of significant electrolyte derangement. We present a case of a 65-year-old male with hypertension and dyslipidemia who presented with transient acute attacks of profound weakness and presyncope, without loss of consciousness or other focal neurologic signs. Initial evaluation, including cardiac, neurologic, and metabolic testing, was unrevealing. Mild hypokalemia (potassium 3.2 mmol/L) was noted on admission. Further endocrine workup revealed a suppressed plasma renin activity (0.5 ng/mL/hr) and elevated plasma aldosterone concentration (28.3 ng/dL) with a high plasma aldosterone concentration/plasma renin activity (PAC/PRA) ratio (56.6), suggestive of PA. Imaging identified two left adrenal gland nodules (4 mm and 6 mm) consistent with adenomas, and adrenal venous sampling demonstrated left-sided predominance. The patient underwent a unilateral retroperitoneoscopic adrenalectomy with complete resolution of symptoms. Prior to surgery, he was treated with amlodipine 10 mg daily and prazosin 1 mg twice daily for blood pressure control. Following adrenalectomy, he maintained normotension without antihypertensive therapy, which persisted at five-year follow-up. This case highlights a rare presentation of PA with episodic severe muscle weakness in the absence of profound hypokalemia. It underscores the importance of considering PA in patients with unexplained episodic weakness and resistant or moderate-to-severe hypertension, even when potassium levels are within normal or mildly low ranges.

## Introduction

Primary aldosteronism (PA) is a frequently overlooked cause of secondary hypertension, accounting for an estimated 5% to 20% of cases [1]. Although classically associated with hypokalemia, the majority of patients present with normokalemia because compensatory mechanisms (such as increased distal sodium delivery and enhanced potassium secretion) are often balanced by dietary potassium intake and renal adaptation. This can often lead to delayed diagnosis [2]. PA is associated with increased cardiovascular morbidity and poorer quality of life compared to essential hypertension [3]. PA increases cardiovascular risk through mechanisms beyond hypertension, including direct effects of aldosterone on vascular inflammation, endothelial dysfunction, arterial stiffness, and cardiac fibrosis. These changes promote atherosclerosis, arrhythmias (notably atrial fibrillation), and heart failure, independent of blood pressure levels. While neuromuscular manifestations such as muscle weakness are typically observed only in the setting of profound hypokalemia, we present a rare case of PA manifesting as episodic, severe muscle weakness with abrupt loss of postural tone, rendering him unable to stand despite only mildly reduced potassium levels. A literature review did not identify any other reported cases of muscle weakness with normokalemia in PA.

## Case presentation

A 65-year-old male with a history of hypertension and dyslipidemia presented to the emergency room with a chief complaint of presyncope. While in his kitchen, he experienced what he termed a ‘jolt’- a sudden extreme weakness associated with presyncope without any loss of consciousness. His children and nephew assisted in lowering him to the floor. He subsequently felt generalized parasthesias for two to three minutes and developed a severe headache. He was transported to the emergency room, where he was found to be in normal sinus rhythm with an initial blood pressure of 167/92 mmHg and a pulse of 92 beats per minute. He was afebrile with an oxygen saturation of 99%. 

Initial laboratory results were significant for a sodium of 142 mmol/L, potassium of 3.2 mmol/L, creatinine of 0.7 mg/dL, serum carbon dioxide 27 mmol/L, magnesium 1.9 mg/dL, B-type natriuretic peptide (BNP) of 59 pg/mL, hemoglobin of 15 g/dL, white blood cell count of 4.7 x 10^3^/uL, and a plasma alcohol level of < 3 mg/dL (Table 1).

**Table 1:** Initial laboratory test results

Test	Result	Units	Reference Range	Interpretation
Sodium	142	mmol/L	135–145	Normal
Potassium	3.2	mmol/L	3.5–5.0	Low
Creatinine	0.7	mg/dL	0.6–1.3	Normal
Carbon Dioxide (CO₂, Serum Bicarbonate)	27	mmol/L	22–29	Normal
Magnesium	1.9	mg/dL	1.7–2.4	Normal
BNP (B-type Natriuretic Peptide)	59	pg/mL	<100	Normal
Hemoglobin	15	g/dL	13.5–17.5 (M) / 12.0–15.5 (F)	Normal
White Blood Cell Count	4.7 ×10³/µL	×10³/µL	4.0–10.5	Normal
Plasma Alcohol Level	<3	mg/dL	<10	Negative

The patient was admitted and ruled out for an acute coronary syndrome with serial negative troponins. Telemetry monitoring showed no significant arrhythmias, and orthostatic blood pressures were normal. A chest CT angiogram was negative for pulmonary embolism, and a lower extremity ultrasound ruled out deep venous thrombosis. He was treated with sumatriptan for his presumptive migraine headaches and was subsequently discharged home. 

**Figure 1:**
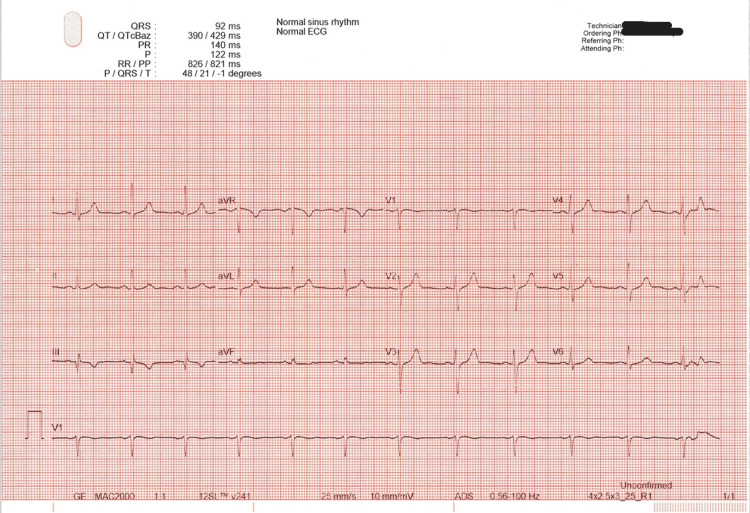
Electrocardiogram (ECG) of the patient

Following his discharge, he underwent an outpatient evaluation by Cardiology for what was labeled as presyncope. He reported multiple episodes over the preceding four weeks of progressive fatigue followed by sudden, extreme weakness resulting in abrupt loss of postural tone, rendering him unable to stand. During these episodes, he was unable to respond or move but typically recovered within 5-10 minutes. During the visit, he was witnessed by the cardiologist to experience an episode of acute weakness in the exam room, slumping to one side, and collapsing, but he remained conscious. The episode lasted for 30 seconds, after which he fully recovered. There were no tonic-clonic movements, incontinence, or tongue biting. The patient was fully aware of the episode, and there was no post-ictal state. He denied symptoms such as diplopia, dysphagia, dysarthria, aphasia, ataxia, diaphoresis, or palpitations. An ECG demonstrated a normal sinus rhythm with normal intervals and normal QTc 429 msec (Figure 1).

Capillary blood glucose measured by fingerstick at the time of his episode was within normal limits. Repeat laboratory workup, including a complete metabolic panel, complete blood count, and TSH, was normal. His potassium level was 3.9 mmol/L. An echocardiogram revealed normal left ventricular size and function, with an ejection fraction of 55-60% and no valvular abnormalities. A two-week event monitor was unremarkable, with patient-triggered events correlating with a normal sinus rhythm. A coronary CT angiogram demonstrated no flow-limiting stenotic disease of the coronary arteries.

Neurology evaluation, including electromyography, nerve conduction studies, electroencephalography, and brain MRI, was normal. Given his hypertension and initial hypokalemia on presentation, the Cardiologist ordered plasma metanephrines, renin, and aldosterone levels. Plasma and 24-hour urine metanephrine levels were within normal limits. The aldosterone-renin ratio (ARR) was obtained under guideline-concordant pre-analytic conditions, including mid-morning blood draw after ≥2 hours upright and 15 minutes seated, with normal dietary sodium intake and serum potassium normalized prior to sampling. The patient was not on a mineralocorticoid receptor antagonist prior to sampling. The plasma renin activity (PRA) measured 0.5 ng/mL/hr (reference interval while upright: 0.5-4.0 ng/mL/hr), the plasma aldosterone concentration (PAC) was 28.3 ng/dL (reference interval while upright: 4.0-31.0 ng/dL), and the PAC/PRA ratio was 56.6 (Table 2).

**Table 2:** Hormonal evaluation for primary aldosteronism *Interpretive guideline: A plasma aldosterone concentration (PAC) > 10 ng/dL, plasma renin activity (PRA) < 1 ng/mL/hr, and PAC/PRA ratio > 20 are suggestive of primary aldosteronism (PA). Plasma renin and aldosterone were measured in the morning after the patient had been seated for 30 minutes.

Test	Patient Value	Reference Range (Upright)	Units
Plasma renin activity (PRA)	0.5	0.5–4.0	ng/mL/hr
Plasma aldosterone concentration (PAC)	28.3	4.0–31.0	ng/dL
PAC/PRA ratio	>20	>20 (suggestive of PA)*	—

**Figure 2:**
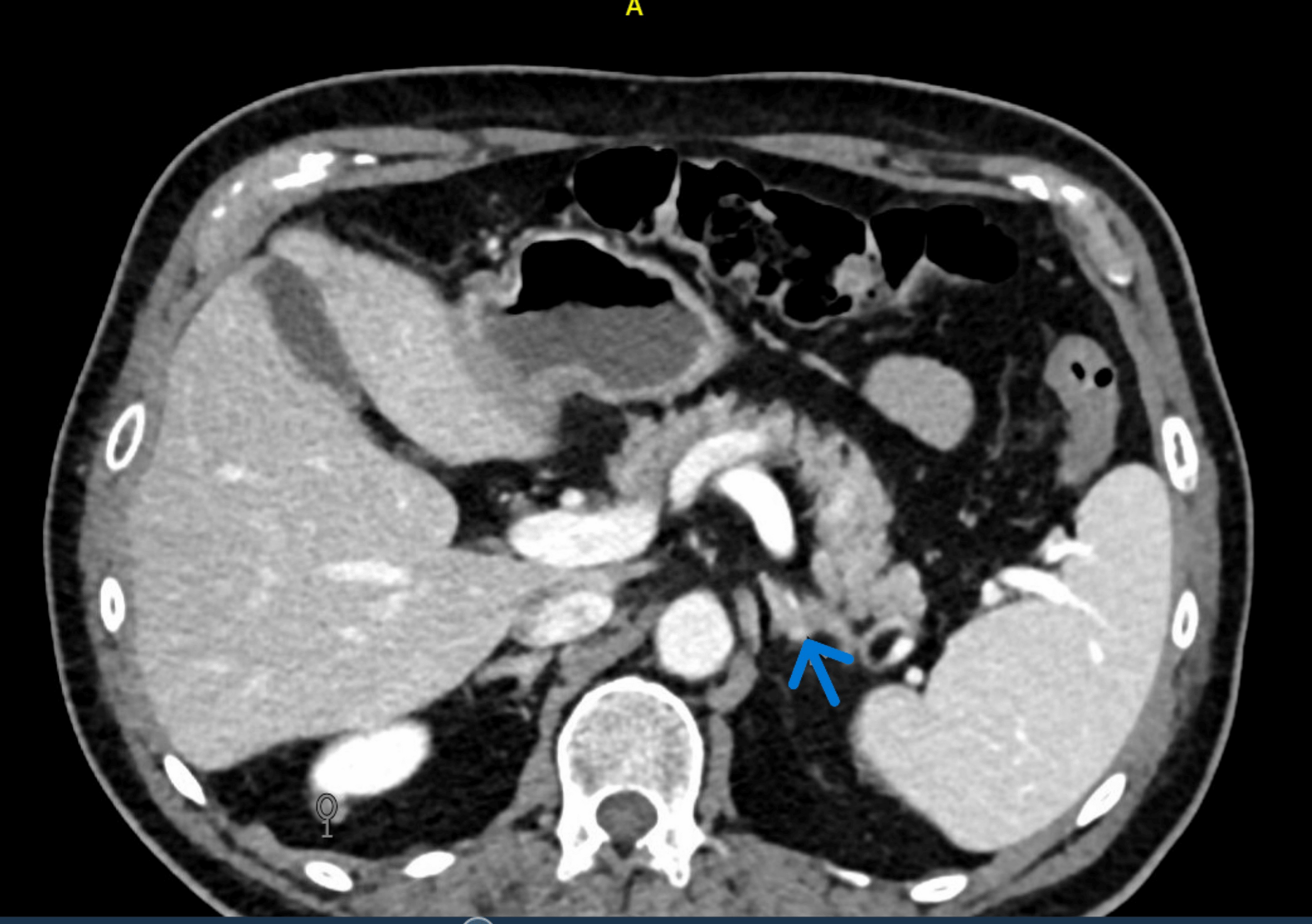
CT image of abdomen Left adrenal nodules observed in the CT image (blue arrow).

Given the PRA < 1, PAC > 10, and PAC/PRA ratio > 20, an abdominal CT scan was performed, revealing two left adrenal gland nodules (4 mm and 6 mm) (Figure 2).

The patient was started on spironolactone 50 mg twice a day, leading to symptomatic improvement. He was later evaluated by Endocrinology and taken off spironolactone in preparation for eventual adrenal venous sampling. Two months after discontinuing spironolactone, he underwent oral salt loading for three days followed by additional testing. His post-salt-load urine sodium level was 219 mmol/24 hrs (reference range: 30-260 mmol/24h), and urine aldosterone was 9.8 mcg/24 hours (reference range: 2.3-21.0 mcg/24 hours). Adrenal vein sampling (AVS) demonstrated adequate cannulation of both adrenal veins, with selectivity indices of 17.4 (left) and 15.0 (right), confirming successful sampling. Post-cosyntropin stimulation at a dose of 250 mcg, aldosterone levels were markedly elevated bilaterally (left: 705 ng/dL; right: 270 ng/dL) compared with peripheral levels (19 ng/dL). The lateralization index (left/right aldosterone-to-cortisol ratio) was 2.6, indicating bilateral aldosterone secretion with left-sided predominance. The lateralization index of 2.6 raised the possibility of bilateral adrenal hyperplasia. Despite the lateralization index falling below 4, a decision was made to proceed with left retroperitoneoscopic adrenalectomy instead of bilateral adrenalectomy to avoid lifelong steroid dependence. Postoperatively, the patient recovered well with freedom from recurrence of any episodic weakness and spontaneous collapse. Postoperatively, morning plasma renin activity measured 0.3 ng/mL/hr and aldosterone 4.6 ng/dL, consistent with biochemical resolution of hyperaldosteronism. At nine-month follow-up, plasma renin activity remained 0.3 ng/mL/hr, with an aldosterone level of 9.4 ng/dL, and serum potassium was 4.5 mEq/L. Prior to surgery, he was treated with amlodipine 10 mg daily and prazosin 1 mg twice daily for blood pressure control. Following adrenalectomy, he maintained normotension without antihypertensive therapy, which persisted at five-year follow-up.

## Discussion

Primary hyperaldosteronism (PHA) is a common cause of hypertension, thought to account for roughly 5-20% of hypertension cases [1]. While classically associated with hypokalemia, studies indicate that hypokalemia is present in only 9-37% of cases [2]. Other associated findings of PHA include an increased risk of cardiovascular events compared with essential hypertension. Specifically, patients with primary aldosteronism have an increased risk of stroke (odds ratio (OR) 2.58, coronary artery disease (OR 1.7), atrial fibrillation (OR 3.52), and heart failure (OR 2.05) [3]. This may in part be explained by the observation that type 2 diabetes and metabolic syndrome are significantly more prevalent in patients with primary hyperaldosteronism than in controls matched for sex, age, BMI, and blood pressure [4]. In addition, evidence shows that when compared with the general population (including those with hypertension), patients with hyperaldosteronism have poorer health-related quality of life. This includes increased anxiety, demoralization, stress, depression, and nervousness, as well as impaired sleep quality [5]. A final, but less common, clinical manifestation of hyperaldosteronism is muscle weakness. This is most commonly associated with serum potassium concentrations below 2.5 meq/L [6].

Given the significant clinical implications, PHA should be considered in all patients with hypertension with hypokalemia, severe hypertension (i.e. blood pressure above 150/100), resistant hypertension (i.e. inadequately controlled blood pressure despite being on three antihypertensive agents including a diuretic), hypertension with adrenal incidentaloma, and onset of hypertension at a young age (i.e. age less than 30) [7]. Initial diagnostic testing should begin with measuring the plasma aldosterone concentration (PAC) and plasma renin activity (PRA). In cases of hyperaldosteronism, the PRA is usually <1 ng/mL per hour, and the PAC is usually >10 ng/dL. The PAC/PRA ratio is usually >20 ng/dL per ng/mL/hour. Of note, the PAC and PRA should be checked at 8 AM with the patient in a seated position. If the PAC and PRA results are suggestive of hyperaldosteronism, then the next step is confirmatory testing. However, in patients with either spontaneous hypokalemia, low PRA, and a PAC ≥20 ng/dL, or with low PRA or PRC and a PAC >30 ng/dL, no confirmatory testing is necessary as these patients are confirmed to have primary hyperaldosteronism [8]. Otherwise, confirmation testing most often consists of oral salt loading. This is typically done by administering 2 g of sodium chloride three times a day for three days. On the third day of sodium loading, serum electrolytes are measured, and a 24-hour urine specimen is obtained. Positive testing consists of 24-hour urine sodium excretion above 200 mEq (4600 mg) to document adequate sodium loading and urine aldosterone excretion of greater than 12 mcg/24 hours. 

Once a diagnosis of primary hyperaldosteronism has been confirmed, it is incumbent on the physician to differentiate between the two most common causes of hyperaldosteronism: a unilateral aldosterone-secreting adenoma versus bilateral hyperplasia. This is done through a combination of CT imaging and adrenal venous sampling [9]. CT adrenal scanning can help identify adrenal masses or nodules, which may help direct surgical therapy. However, in order to confirm an aldosterone-secreting adenoma, it is typically necessary to perform adrenal venous sampling. The exception to this is when younger patients less than age 35 are found to have an adrenal nodule greater than 1 cm with hypokalemia and a plasma aldosterone concentration of greater than 30 ng/dL (i.e., incontrovertible hyperaldosteronism). Otherwise, adrenal sampling is required to differentiate between bilateral adrenal hyperplasia and an aldosterone-secreting adenoma. Typically, aldosterone concentrations found on adrenal venous sampling will be relatively similar between the right and left adrenal glands in cases of bilateral hyperplasia (which accounts for approximately 60% of cases). In contrast, an aldosterone-secreting adenoma will typically demonstrate aldosterone sampling four times greater than the contralateral adrenal gland. The distinction between hyperplasia and an adenoma is important because the treatment of the two differs. In cases of adenoma, hyperaldosteronism can be treated and often cured with unilateral adrenalectomy. Whereas bilateral adrenal hyperplasia is treated medically with aldosterone suppression using a mineralocorticoid receptor antagonist. 

In our case, the patient’s PRA and PAC levels and his oral salt loading results helped confirm hyperaldosteronism. Given his CT scan results and adrenal venous sampling (approaching a lateralization index of 4), a decision was made to proceed with unilateral left adrenalectomy. Fortunately, the patient responded very well with resolution of his presenting complaint of intermittent profound muscle weakness with collapse and concomitant resolution of his hypertension. He was able to discontinue all antihypertensives post-op and maintain a normal blood pressure. At five years post-surgery, he has remained symptom-free and normotensive.

Our case represents a rare presentation of hyperaldosteronism manifesting as profound muscle weakness. A comprehensive literature search yielded only a handful of cases where profound muscle weakness served as the presenting symptom of hyperaldosteronism [10-12]. In each and every one of these cases, the patients had profound hypokalemia on presentation. Our case is highly unusual in that the potassium levels were only very mildly reduced on the initial presentation and were unremarkable on subsequent checks, including when the patient was symptomatic. Typically, one would expect potassium levels below 2.5 mEq/L before observing muscle weakness. In our case, the potassium on initial presentation was 3.2 mEq/L. Subsequent measurements were consistently above 3.5 mEq/L. This raises the question of whether there may have been some additional mechanism behind his acute loss of muscle tone. This might include an underlying myopathy that was subclinical and only manifested with mild periodic hypokalemia. Although hypokalemia is the primary driver of myopathy in hyperaldosteronism, additional mechanisms-including magnesium depletion, metabolic alkalosis, and direct aldosterone-mediated skeletal muscle catabolism and oxidative injury-may also contribute to muscle weakness. Notably, our patient has not had any recurrence of his episodes for five years since his adrenalectomy, which seems to rule out an underlying pathology unrelated to his hyperaldosteronism.

## Conclusions

Hyperaldosteronism is an underrecognized cause of hypertension. It is associated with an increased risk of cardiovascular events compared with essential hypertension and is also associated with impaired quality of life. These associated risks point to pathophysiological effects of hyperaldosteronism above and beyond the negative impact of hypertension alone. Our case report highlights an unusual case of severe episodic muscle weakness that was not associated with profound hypokalemia. This raises the question of whether hyperaldosteronism may have direct impacts on neuromuscular pathology beyond hypokalemia alone.
